# *Salmonella* Uses Energy Taxis to Benefit from Intestinal Inflammation

**DOI:** 10.1371/journal.ppat.1003267

**Published:** 2013-04-18

**Authors:** Fabian Rivera-Chávez, Sebastian E. Winter, Christopher A. Lopez, Mariana N. Xavier, Maria G. Winter, Sean-Paul Nuccio, Joseph M. Russell, Richard C. Laughlin, Sara D. Lawhon, Torsten Sterzenbach, Charles L. Bevins, Renée M. Tsolis, Rasika Harshey, L. Garry Adams, Andreas J. Bäumler

**Affiliations:** 1 Department of Medical Microbiology and Immunology, School of Medicine, University of California, Davis, Davis, California, United States of America; 2 Department of Veterinary Pathobiology, College of Veterinary Medicine and Biomedical Sciences, Texas A&M University, College Station, Texas, United States of America; 3 Section of Molecular Genetics and Microbiology, School of Biological Sciences, University of Texas at Austin, Austin, Texas, United States of America; Stanford University School of Medicine, United States of America

## Abstract

Chemotaxis enhances the fitness of *Salmonella enterica* serotype Typhimurium (*S.* Typhimurium) during colitis. However, the chemotaxis receptors conferring this fitness advantage and their cognate signals generated during inflammation remain unknown. Here we identify respiratory electron acceptors that are generated in the intestinal lumen as by-products of the host inflammatory response as *in vivo* signals for methyl-accepting chemotaxis proteins (MCPs). Three MCPs, including Trg, Tsr and Aer, enhanced the fitness of *S*. Typhimurium in a mouse colitis model. Aer mediated chemotaxis towards electron acceptors (energy taxis) *in vitro* and required tetrathionate respiration to confer a fitness advantage *in vivo*. Tsr mediated energy taxis towards nitrate but not towards tetrathionate *in vitro* and required nitrate respiration to confer a fitness advantage *in vivo*. These data suggest that the energy taxis receptors Tsr and Aer respond to distinct *in vivo* signals to confer a fitness advantage upon *S*. Typhimurium during inflammation by enabling this facultative anaerobic pathogen to seek out favorable spatial niches containing host-derived electron acceptors that boost its luminal growth.

## Author Summary

The ability to thrive in the inflamed gut is crucial for *Salmonella* serotypes to ensure their transmission to a new host. *Salmonella* serotypes can use alternative electron acceptors that become available during colitis to support their growth by anaerobic respiration, therefore giving the pathogen an advantage over the fermenting microbiota. While it is known that *S*. Typhimurium benefits from chemotaxis and motility during colitis, the chemotaxis signals the pathogen detects *in vivo* have not been elucidated. Here we demonstrate that during intestinal inflammation, *S*. Typhimurium is able to seek energetically favorable niches by detecting the presence of nitrate and tetrathionate, two respiratory electron acceptors that are generated as by-products of the host inflammatory response. Through this mechanism, energy taxis provides a fitness advantage for *S*. Typhimurium, and likely other facultative anaerobic bacteria, during *in vivo* growth in the intestinal lumen.

## Introduction

The enteric pathogen *S.* Typhimurium uses its virulence factors, two type III secretion systems (T3SS-1 and T3SS-2), to trigger acute intestinal inflammation [1]. Interestingly, the pathogen can benefit from the host inflammatory response, as indicated by its enhanced luminal growth during colitis [2], [3]. The resulting bloom of *S.* Typhimurium in the lumen of the inflamed gut enhances its rate of transmission [4]. However, the properties that enhance the fitness of *S.* Typhimurium during colitis remain incompletely understood.

Flagella-mediated motility is required for efficient growth of *S.* Typhimurium in the lumen of the inflamed intestine [5], but not in the absence of intestinal inflammation [6], suggesting that the inflammatory host response creates an environment that favors the growth of motile bacteria. The fitness advantage conferred by flagella in the gut is dependent on chemotaxis [6], a property that is predicted to enable *S.* Typhimurium to seek out favorable metabolic niches. It has been proposed that motility and chemotaxis enable *S.* Typhimurium to migrate towards complex polysaccharides available for fermentation. Galactose residues are present in high abundance at the cecal mucosa [6] and might attract *S.* Typhimurium through Trg, a methyl-accepting chemotaxis protein (MCP) that senses galactose and ribose [7], [8], [9]. Chemotaxis is required for *S.* Typhimurium to get close to the mucosal surface and it has been hypothesized that a concentration gradient of galactose residues might be a possible chemotactic cue that attracts *S.* Typhimurium towards the intestinal epithelium [6]. However, there is currently no direct evidence to support the proposition that chemotaxis towards fermentable sugars allows *S.* Typhimurium to enhance its growth during intestinal inflammation. Mucus production in the gut is raised as part of the mucosal defense against *S.* Typhimurium infection [6], [10], which is consistent with the idea that the availability of fermentable sugars becomes elevated during infection. On the other hand, mucus production is not abrogated in the absence of intestinal inflammation. Furthermore, no chemotactic signals that are absent in the non-inflamed gut, but generated during inflammation have been identified.

We thus reasoned that further investigation into the mechanisms by which chemotaxis enhances the fitness of *S.* Typhimurium during colitis was warranted. To this end, we determined how mutations in genes encoding different MCPs affect growth in the inflamed intestine and performed follow-up studies to elucidate the mechanisms by which chemotaxis confers a growth advantage during intestinal inflammation.

## Results

### Trg, Aer and Tsr confer a competitive advantage in the mouse colitis model

We used the mouse colitis model [11] to investigate why chemotaxis provides an advantage in the environment of the inflamed gut. In this model, mice are inoculated with streptomycin one day prior to infection with *S.* Typhimurium. Preconditioning with streptomycin disrupts the resident microbiota and allows *S*. Typhimurium to elicit acute cecal inflammation, which is an animal model for human gastroenteritis (reviewed in [12]). We reasoned that a first step in elucidating the mechanism by which chemotaxis increases the fitness of *S.* Typhimurium during inflammation would be to determine which MCP(s) confer(s) a luminal growth advantage in the mouse colitis model. To this end we generated *S.* Typhimurium strains carrying mutations in *mcpA* (FR37), *mcpB* (FR36), *mcpC* (FR35), *trg* (FR42), *aer* (FR5) or *tsr* (FR4). As a positive control, we included a chemotaxis-deficient *S*. Typhimurium *cheY* mutant (FR13), which lacks a core chemotaxis component that is essential for signal transduction from MCPs to the flagellar apparatus. Each mutant was tested for its ability to compete with wild-type *S.* Typhimurium (IR715) for luminal growth in the mouse colitis model (competitive infection assay).

**Figure 1:**
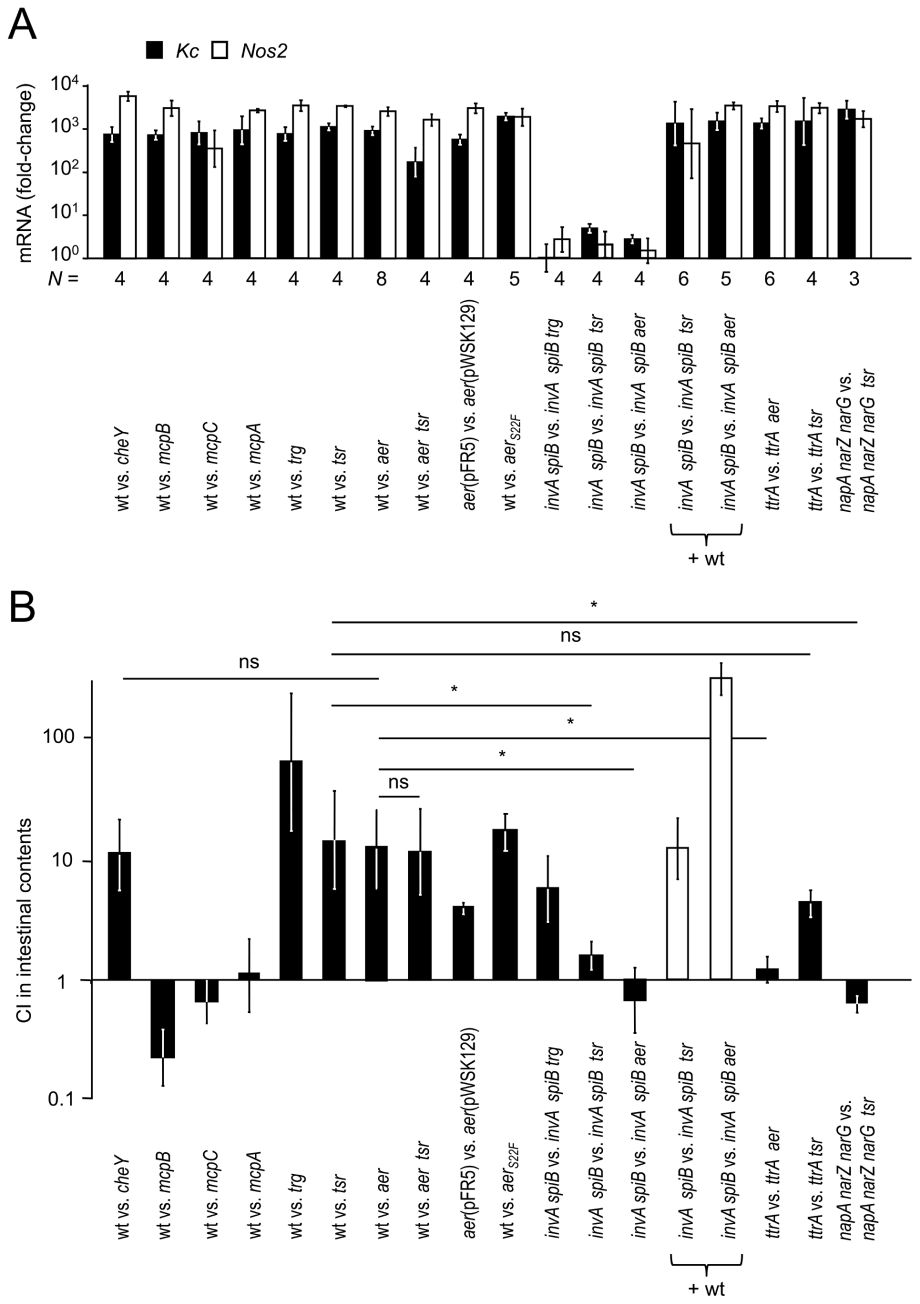
The *aer* and *tsr* genes confer a competitive growth advantage during colitis. Groups of streptomycin pre-treated mice (*N* is indicated below the graph in panel A) were inoculated with equal mixtures of the indicated *S*. Typhimurium strains and organs were collected for analysis four days after infection. Mice were infected either with an equal mixture of two strains (black bars in panel B) or with an equal mixture of the *S*. Typhimurium wild type (+ wt) and two mutant strains (white bars in panel B). (A) Induction of transcripts encoding the inflammatory markers Kc (encoded by the *Kc* gene, black bars) and iNOS (encoded by the *Nos2* gene, white bars) in the murine cecum after infection with the indicated mixtures of *S.* Typhimurium strains. Expression of *Kc* and *Nos2* in the cecal mucosa was determined by quantitative real-time PCR analysis. Bars represent geometric means of *Kc* and *Nos2* mRNA copy numbers as fold-change over mRNA levels in mock-infected mice ± standard error. (B) Competitive indices (CI) of *S*. Typhimurium strains recovered from the colon contents of infected mice. Bars represent geometric means ± standard error. *, *P*<0.05; ns, not significant.

Infection of streptomycin pre-treated mice with the *S*. Typhimurium wild type (IR715) and one of the above mutants resulted in acute cecal inflammation (Fig. S1) and markedly increased cecal mRNA levels of the *Kc* and *Nos2* genes (Fig. 1A). *Kc* and *Nos2* encode the inflammatory markers keratinocyte-derived cytokine (KC) and inducible nitric oxide synthase (iNOS), respectively. Wild-type *S*. Typhimurium (IR715) was recovered in significantly higher numbers (*P*<0.05) from the colon contents than a chemotaxis-deficient *cheY* mutant (FR13) four days after infection (Fig. 1B), which confirmed previous observations in this model [6]. Consistent with the prediction that chemotaxis towards galactose residues might enhance the fitness of *S.* Typhimurium in the inflamed gut [6], the *trg* mutant (FR42) was recovered in lower numbers than the wild type. In contrast, mutations in *mcpA, mcpB* or *mcpC* did not reduce the fitness of *S.* Typhimurium (Fig. 1B). Remarkably, the *S.* Typhimurium wild type (IR715) was recovered in significantly (*P*<0.05) higher numbers from colon contents than an *aer* mutant (FR5) or a *tsr* mutant (FR4) (Fig. 1B), which identified Tsr and Aer as two new MCPs required for luminal growth during colitis. The growth advantage of wild-type *S.* Typhimurium (IR715) over an *aer tsr* mutant (FR6) was not significantly greater than that over a *tsr* mutant (FR4).

In *S.* Typhimurium, *aer* is located upstream of *mcpC*, a gene encoding a MCP that is absent from *Escherichia coli* [13], [14] (Fig. S2A), thereby raising the possibility that an insertion in *aer* could be polar on expression of the downstream *mcpC* gene. However, inactivation of *aer* did not alter *mcpC* mRNA levels *in vitro* (Fig. S2B), which made it unlikely that polar effects on *mcpC* could account for the phenotype of the *aer* mutant (FR5) in the mouse colitis model (Fig. 1B). Mutational inactivation of *mcpC* did not reduce fitness (Fig. 1B), suggesting that Aer, but not McpC, provides a competitive luminal growth advantage in the mouse colitis model. Furthermore, we cloned the *aer* gene under the control of its native promoter on a low-copy number plasmid (pFR5) and introduced this plasmid into the *aer* mutant. Streptomycin pre-treated mice were infected with an equal mixture of the *aer* mutant carrying a control vector (pWSK129) and an *aer* mutant complemented with the cloned *aer* gene cloned on pFR5. The complemented *aer* mutant (*aer*[pFR5]) was recovered in significantly (*P*<0.05) higher numbers from colon contents than the *aer* mutant carrying a control vector (*aer*[pWSK129]) (Fig. 1B), thereby providing strong support for the idea that Aer confers a growth benefit in the mouse colitis model.

*S*. Typhimurium causes intestinal inflammation by using T3SS-1 for epithelial invasion and T3SS-2 for macrophage survival [1]. Inactivation of T3SS-1 (through a mutation in *invA*) and T3SS-2 (through a mutation in *spiB*) renders *S*. Typhimurium unable to trigger gut inflammation in the mouse colitis model [10]. To investigate whether migration towards galactose residues would confer a luminal growth advantage in the absence of intestinal inflammation, streptomycin pre-treated mice were infected with an equal mixture of an *invA spiB* mutant (SPN452) and an *invA spiB trg* mutant (FR43). Mice infected with this mixture did not exhibit elevated expression of inflammatory markers (Fig. 1A) and the *invA spiB trg* mutant was recovered in lower numbers than the *invA spiB* mutant (Fig. 1B), suggesting that Trg conferred a luminal growth advantage in the absence of intestinal inflammation. To determine whether the *aer* gene and the *tsr* gene conferred a fitness advantage in the absence of intestinal inflammation, streptomycin pre-treated mice were infected with an equal mixture of an *invA spiB* mutant (SPN452) and an *invA spiB aer* mutant (FR11) or an *invA spiB* mutant (SPN452) and an *invA spiB tsr* mutant (FR10). Mice infected with these mixtures neither developed intestinal pathology (Fig. S1) nor exhibited elevated expression of inflammatory markers (Fig. 1A). An equal recovery of both strains from colon contents (Fig. 1B) suggested that neither Aer nor Tsr boosted luminal growth of *S.* Typhimurium in the absence of intestinal inflammation.

To confirm that equal recovery of an *invA spiB* mutant and an *invA spiB aer* mutant from mice was due to lack of inflammation, we performed an additional experiment in which inflammation was restored by adding wild-type *S.* Typhimurium. To this end, streptomycin pre-treated mice were infected with a mixture of the *S.* Typhimurium wild type (ATCC14028), an *invA spiB* mutant (SPN452) and an *invA spiB aer* mutant (FR11). Mice infected with this mixture developed acute intestinal inflammation, as indicated by elevated expression of inflammatory markers (Fig. 1A). The *invA spiB* mutant was recovered in higher numbers than the *invA spiB aer* mutant (Fig. 1B), suggesting that inflammation induced by the *S.* Typhimurium wild type generated an environment in which the *aer* gene conferred a fitness advantage upon the *invA spiB* mutant. Similar results were obtained when mice were infected with the *S.* Typhimurium wild type (ATCC14028), an *invA spiB* mutant (SPN452) and an *invA spiB tsr* mutant (FR10) (Fig. 1A and 1B). Collectively, these data supported the working hypothesis that signals generated by the host inflammatory response are detected through Aer and Tsr, thereby enabling *S*. Typhimurium to migrate towards favorable luminal niches to boost its growth. However, the nature of these signals remained obscure.

### Aer mediates energy taxis towards tetrathionate

Enhanced luminal growth of *S.* Typhimurium during colitis has been linked to the transmigration of neutrophils into the intestinal lumen [15]. Neutrophils release reactive oxygen species (ROS) in an attempt to kill bacteria. A by-product of releasing ROS is the oxidation of endogenous sulfur compounds, such as thiosulfate (S_2_O_3_^2−^) generated by the colonic epithelium [16], [17], to tetrathionate (S_4_O_6_^2−^) [18]. Since tetrathionate becomes available only during inflammation [18], we investigated whether this compound could serve as a signal for Aer and/or Tsr during colitis. We tested this hypothesis using the motility plate assay, in which formation of a halo by migration of *S.* Typhimurium from a point of inoculation into the surrounding motility agar was measured after anaerobic incubation for 6.5 hours. Halo formation was not observed with non-motile (*flgK* mutant, SW215) or chemotaxis-deficient (*cheY* mutant, FR13) strains. The *tsr* mutant (FR4) formed halos with significantly (*P*<0.05) reduced diameter compared to those produced by the *S.* Typhimurium wild type (IR715) both in the presence or in the absence of tetrathionate (Fig. S3), suggesting that Tsr responded to signals other than (or in addition to) tetrathionate in this assay. In contrast, the wild type (IR715) formed halos with a significantly (*P*<0.05) larger diameter than an *aer* mutant (FR5) when tetrathionate was present in the motility agar, but not when tetrathionate was absent (Fig. S3A and S3B). Anaerobic growth in LB broth inoculated with the wild type (IR715) and an *aer* mutant (FR5) or the wild type and a *tsr* mutant (FR4) indicated that inactivation of these chemotaxis receptors did not produce a growth defect (Fig. S3B). These data supported the idea that Aer mediates chemotaxis towards tetrathionate.

Since tetrathionate is not known to bind Aer directly, we were interested in the mechanism by which Aer could detect this compound. The *ttrBCA* gene cluster encodes a tetrathionate reductase that enables *S.* Typhimurium to oxidize NADH/H^+^ by transferring electrons from this donor through the electron transport chain to the terminal electron acceptor tetrathionate (tetrathionate respiration) [19]. Aer possesses a flavin adenine dinucleotide (FAD)-binding domain that enables this MCP to respond to changes in the redox status of the bacterial cell [20], [21], a process termed energy taxis [22]. We thus reasoned that by reducing tetrathionate, the TtrBCA tetrathionate reductase might change the redox status of the bacterial cell, thereby generating a signal that is sensed by Aer. This hypothesis would predict that a tetrathionate respiration-deficient mutant would be unable to migrate towards tetrathionate by Aer-mediated energy taxis. To test this prediction, we assessed the ability of a tetrathionate respiration-deficient (*ttrA*) mutant to migrate towards tetrathionate in the motility plate assay. Consistent with our hypothesis, a *ttrA* mutant (SW661) formed halos with a significantly (*P*<0.05) smaller diameter than the wild type (IR715) when tetrathionate was present in the motility agar, but not when this terminal electron acceptor was absent (Fig. S3A and S3B).

**Figure 2:**
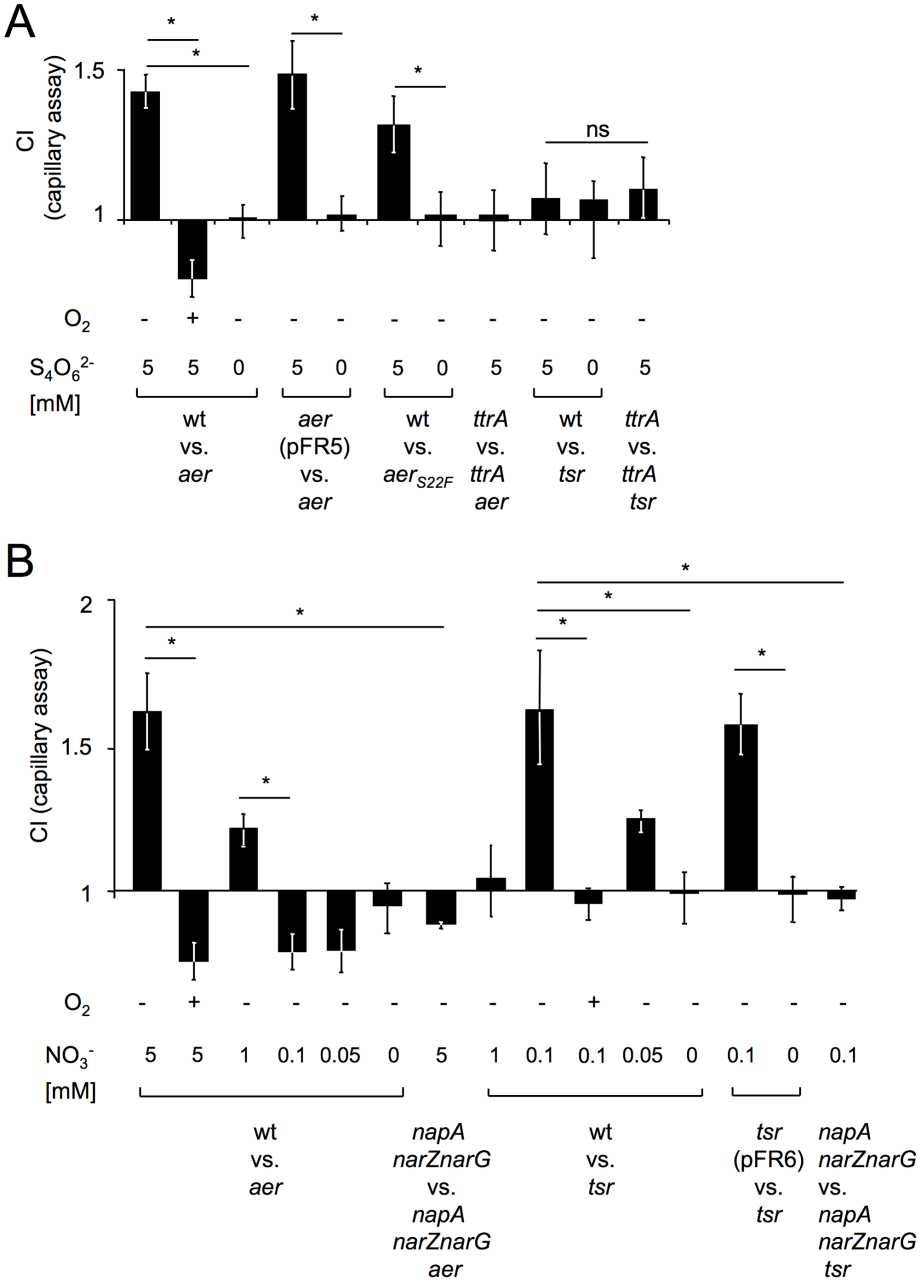
The *aer* and *tsr* genes mediate energy taxis towards tetrathionate and nitrate, respectively. Migration of the indicated *S.* Typhimurium strains from a reservoir into a capillary was measured using the capillary assay. The competitive index was calculated by dividing the ratio of the two competing strains present in the capillary after 45 minutes of incubation through the ratio present in the inoculum. Bars represent geometric means of competitive indices (CI) from at least three independent experiments ± standard error. *, *P*<0.05; ns, not significant; wt, wild type. (A) Migration into a capillary containing the indicated concentrations of tetrathionate (S_4_O_6_^2−^) was determined after incubation under anaerobic conditions (O_2_, −) or in the presence of oxygen (O_2_, +). (B) Migration into a capillary containing the indicated concentrations of nitrate (NO_3_^−^) was determined after incubation under anaerobic conditions (O_2_, −) or in the presence of oxygen (O_2_, +).

To further investigate whether tetrathionate respiration serves as a signal for Aer, we used the capillary assay [23], in which a glass capillary was submerged in a reservoir containing an equal mixture of two *S.* Typhimurium strains and migration into the capillary was measured after a 45 minute anaerobic incubation. In the absence of tetrathionate, the wild type (IR715) and an *aer* mutant (FR5) were recovered in equal numbers from the capillary. In contrast, the wild type was recovered in significantly higher numbers when the capillary contained tetrathionate (Fig. 2A and Table S1). Introduction of the cloned *aer* gene (pFR5) into the *aer* mutant resulted in increased recovery from a capillary containing tetrathionate. These data provided further support for the idea that Aer mediates chemotaxis towards tetrathionate. When a capillary containing tetrathionate was submerged in a reservoir filled with a *ttrA* mutant and a *ttrA aer* mutant, both strains were recovered in equal numbers (Fig. 2A), suggesting that Aer-mediated chemotaxis towards tetrathionate was dependent on tetrathionate respiration *in vitro*. Consistent with the fact that genes for the utilization of inferior electron acceptors are repressed in the presence of oxygen [24], we did not observe energy taxis towards tetrathionate when the assay was repeated under aerobic conditions (Fig. 2A). To test the idea that Aer detects tetrathionate by sensing changes in the redox status of the cell, we generated a strain carrying a point mutation in the *aer* open reading frame (C65T) leading to an amino acid substitution (S22F) that inactivates the FAD-binding domain of Aer (*aer_S22F_* mutant, FR47) [25]. Under anaerobic conditions, the *S.* Typhimurium wild type was recovered in significantly higher numbers than the *aer_S22F_* mutant from a tetrathionate-containing capillary (Fig. 2A), which supported the idea that Aer senses tetrathionate by measuring the redox status of the cell. Interestingly, the wild type (IR715) and a *tsr* mutant (FR4) were recovered in equal numbers from a capillary containing tetrathionate (Fig. 2A), suggesting that Tsr does not mediate taxis towards tetrathionate *in vitro*.

To investigate whether the fitness advantage conferred by the *aer* and/or *tsr* genes was dependent on the ability of *S.* Typhimurium to perform tetrathionate respiration *in vivo*, we infected groups of streptomycin pre-treated mice with equal mixtures of either a tetrathionate respiration-deficient mutant (*ttrA* mutant, SW661) and a *ttrA aer* mutant (FR9) or a *ttrA* mutant (SW661) and a *ttrA tsr* mutant (FR8). The *ttrA* mutant (SW661) outcompeted the *ttrA tsr* mutant (FR8) (Fig. 1B), suggesting that the fitness advantage conferred by Tsr was independent of tetrathionate respiration. In contrast, the fitness advantage conferred by Aer was abrogated during competition of the *ttrA* mutant (SW661) with the *ttrA aer* mutant (FR9). These data suggested that the ability of *S.* Typhimurium to utilize tetrathionate as an electron acceptor was required to see a benefit of Aer during growth in the inflamed gut. Finally, the *S.* Typhimurium wild type outcompeted the *aer_S22F_* mutant (FR47) in streptomycin pre-treated mice, suggesting that redox sensing by Aer conferred a fitness advantage in the mouse colitis model.

To rule out that our phenotype was a result of disrupting the microbiota by treating mice with streptomycin, we infected genetically resistant (CBA) mice in the absence of streptomycin (mouse typhoid model). In this model, mice develop cecal inflammation by day 10 after infection [26]. Mice were infected with an equal mixture of the *S.* Typhimurium wild type (IR715) and an *aer* mutant (FR5) or with an equal mixture of a *ttrA* mutant (SW661) and a *ttrA aer* mutant (FR9). Wild-type *S*. Typhimurium was recovered in significantly higher numbers (*P*<0.05) from colon contents than an *aer* mutant at 10 days and 28 days after infection (Fig. S4). At 10 days after infection, the fitness advantage conferred by Aer was significantly (*P*<0.05) reduced during competition of the *ttrA* mutant with the *ttrA aer* mutant. By day 28 after infection, strains defective for tetrathionate respiration (i.e. the *ttrA* mutant and the *ttrA aer* mutant) were no longer recovered from colon contents.

### Tsr mediates energy taxis towards nitrate

Inflammation induces expression of inducible nitric oxide synthase (iNOS), an enzyme catalyzing the production of nitric oxide (NO). Nitric oxide can react with ROS to generate nitrate (NO_3_^−^), an electron acceptor that becomes available during inflammation [26]. Unlike tetrathionate reductases, nitrate reductases transport protons by scalar chemistry [24]. Since Tsr is proposed to sense changes in the proton-motive force [20], we reasoned that this MCP might sense nitrate during colitis. We used the capillary assay under anaerobic conditions to test this idea. In the absence of nitrate, the wild type (IR715) and a *tsr* mutant (FR4) were recovered in equal numbers from the capillary. However, when the capillary contained 0.1 mM nitrate, the wild type was recovered in significantly higher numbers than the *tsr* mutant (Fig. 2B and Table S1). Introduction of the cloned *tsr* gene (pFR6) into the *tsr* mutant resulted in increased recovery from a capillary containing 0.1 mM nitrate. Interestingly, the wild type was recovered in similar numbers as an *aer* mutant from a capillary containing 0.1 mM nitrate. However, Aer was able to mediate energy taxis towards higher concentrations of nitrate, because the wild type was recovered in significantly higher numbers than an *aer* mutant from a capillary containing 1 mM nitrate. Neither Aer nor Tsr mediated energy taxis towards nitrate when the assay was repeated under aerobic conditions. In conclusion, Tsr, but not Aer, functioned in mediating energy taxis towards low concentrations of nitrate in an anaerobic environment.

*S.* Typhimurium encodes three nitrate reductases, encoded by the *narGHJI*, *narZYWV* and *napFDAGHBC* operons. Inactivation of these three nitrate reductases in a *napA narZ narG* mutant abrogates the ability of *S.* Typhimurium to perform nitrate respiration [26]. A *napA narZ narG* mutant (CAL50) and a *napA narZ narG tsr* mutant (FR46) were recovered in similar numbers from a capillary containing 0.1 mM nitrate (Fig. 2B), suggesting that Tsr-mediated taxis towards nitrate required bacteria to perform nitrate respiration.

To investigate whether the fitness advantage conferred by the *tsr* gene was dependent on the ability of *S.* Typhimurium to perform nitrate respiration *in vivo*, we infected groups of streptomycin pre-treated mice with an equal mixture of a nitrate respiration-deficient mutant (*napA narZ narG* mutant, CAL50) and a *napA narZ narG tsr* mutant (FR46). The fitness advantage conferred by Tsr was abrogated during competition of the *napA narZ narG* mutant with the *napA narZ narG tsr* mutant (Fig. 1B). These data suggested that the ability of *S.* Typhimurium to utilize nitrate as an electron acceptor was required to see a benefit of Tsr during growth in the inflamed gut.

### Energy taxis increases bacterial numbers in close proximity to the mucosal surface

**Figure 3:**
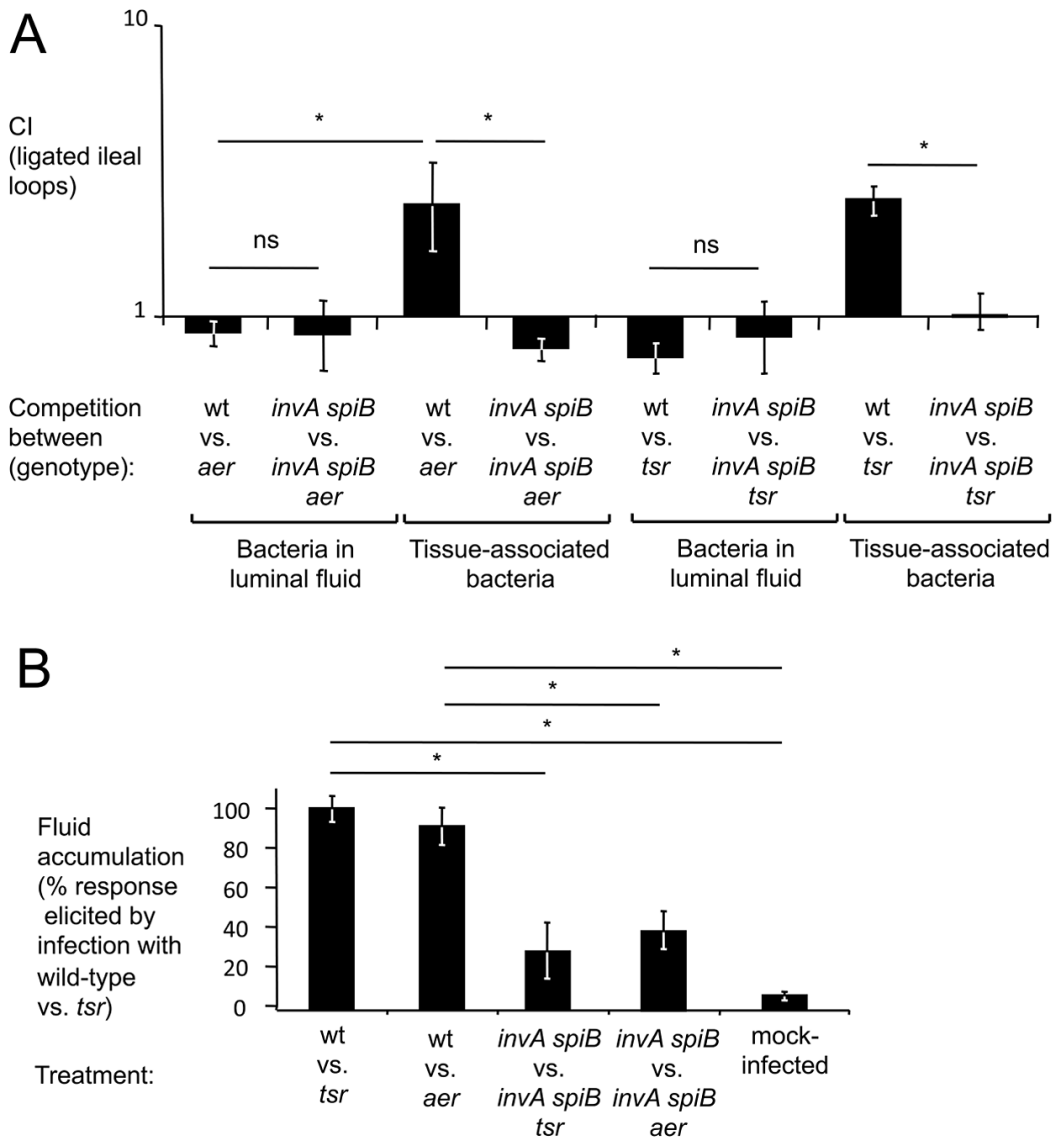
Inactivation of *aer* or *tsr* decreases numbers of *S.* Typhimurium in close proximity to the inflamed mucosal surface. Bovine ligated ileal loops (*N*: each inoculum was tested in loops from 4 different animals) were infected with an equal mixture of the indicated *S*. Typhimurium strains and samples collected 8 hours after infection from the luminal fluid and tissue punches (tissue-associated bacteria). (A) Bars represent geometric means of competitive indices (CI) ± standard error. (B) Fluid accumulation observed 8 hours after infection expressed as a percent of the response observed in loops infected with an equal mixture of the *S*. Typhimurium wild-type strain (AJB715) and a *tsr* mutant. Bars represent geometric means ± standard error. *, *P*<0.05; ns, not significantly different.

Tetrathionate respiration has recently been shown to enhance growth of *S.* Typhimurium in bovine ligated ileal loops [18]. Since bacteria are recovered at a defined early time point (8 hours) after infection, we reasoned that this model would be well suited for chemotaxis experiments. The events leading to the formation of tetrathionate (i.e. the generation of thiosulfate by the colonic epithelium and the respiratory burst of transmigrating neutrophils) are expected to occur in close proximity to the epithelium. To investigate the benefit of tetrathionate respiration in different luminal compartments, we infected bovine ligated ileal loops with an equal mixture of an *aer* mutant (FR5) and a *S*. Typhimurium wild-type strain that was marked by a *phoN*::Kan insertion to facilitate recovery (AJB715) (competitive infection assay). Remarkably, higher numbers (*P*<0.05) of the wild-type strain (AJB715) were found in close association with tissue, while equal numbers of both strains were recovered from the luminal fluid 8 hours after infection (Fig. 3A). No benefit of energy taxis was observed when loops were infected with an equal mixture of an *invA spiB* mutant (SPN452) and an *invA spiB aer* mutant (FR11) (Fig. 3A). Measurements of fluid accumulation, a surrogate for the severity of intestinal inflammation [27], suggested that the severity of inflammatory changes was significantly (*P*<0.05) reduced in loops infected with a mixture of the *invA spiB* mutant and the *invA spiB aer* mutant compared to loops infected with a mixture of the wild type and *aer* mutant (Fig. 3B). The wild type (AJB715) was recovered in higher numbers than a *tsr* mutant (FR4) from tissue-associated samples, but not from the luminal fluid of loops infected with a mixture of both strains (Fig. 3A). We conclude that Tsr and Aer-mediated energy taxis increased bacterial numbers in spatial niches that were in close proximity to the inflamed mucosal surface.

### Energy taxis boosts growth of *S.* Typhimurium in the inflamed intestine

**Figure 4:**
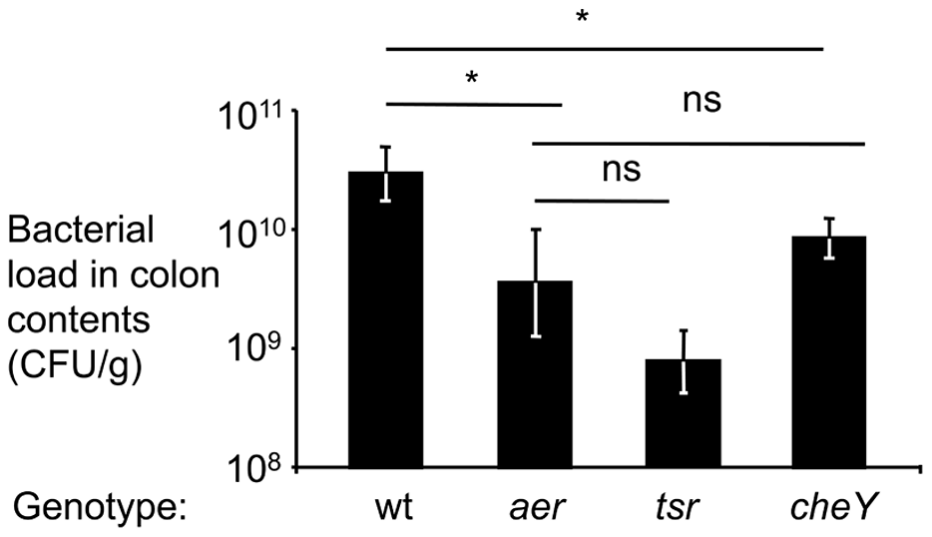
Aer and Tsr boost growth in the mouse colitis model. Groups of streptomycin pre-treated mice were inoculated with the *S*. Typhimurium wild-type strain (wt), an *aer* mutant (*aer*), a *tsr* mutant (*tsr*) or a *cheY* mutant (*cheY*) and colon contents were collected for analysis four days after infection. The numbers of animals (*N*) used in each group is indicated in Figure S4A. Bacterial load in the colon contents of infected mice are shown. Bars represent geometric means ± standard error. *, *P*<0.05; ns, not significant.

Finally, to determine the consequences of energy taxis on bacterial growth after oral infection with individual strains, streptomycin pre-treated mice were inoculated with sterile LB broth (mock infection), the *S.* Typhimurium wild type (IR715), an *aer* mutant (FR5), a *tsr* mutant (FR4) or a chemotaxis-deficient mutant (*cheY* mutant, FR13) and organs were collected for analysis four days later. Mice infected with the *S.* Typhimurium wild type developed acute cecal inflammation (Fig. S5). There was a modest reduction in the severity of cecal inflammation in mice infected with an *aer* mutant or a *cheY* mutant, but the severity of lesions was similar in mice infected with the *tsr* mutant. Importantly, the *aer* mutant, the *tsr* mutant and the *cheY* mutant were recovered in significantly (*P*<0.05) reduced numbers from the colon contents of mice than wild-type *S.* Typhimurium (Fig. 4). Collectively, our data suggested that Tsr and Aer-mediated energy taxis towards electron acceptors generated by the host inflammatory response conferred a fitness advantage in the mouse colitis model (Fig. S6).

## Discussion

Motility and chemotaxis confer a fitness advantage when *S.* Typhimurium resides in the lumen of the inflamed gut, but not during luminal growth in the non-inflamed intestine [6]. One important difference between the non-inflamed and the inflamed large bowel is that growth by anaerobic respiration becomes possible only in the latter niche [18], [26]. Changes in internal energetic conditions that accompany the switch from fermentative growth to growth by anaerobic respiration can be sensed by MCPs, which transduce this signal to the flagella apparatus to enable bacteria to approach spatial niches containing optimal concentrations of respiratory electron acceptors, a process termed energy taxis [22].

Colonic hydrogen sulfide produced by the fermenting microbiota is converted to thiosulfate by the colonic epithelium [16], [17]. During inflammation, neutrophils transmigrate into the intestinal lumen where they release ROS that oxidize thiosulfate to tetrathionate [18]. Furthermore, ROS can react with nitric oxide produced by iNOS to form the electron acceptor nitrate [28]. *S.* Typhimurium readily consumes these electron acceptors to boost its luminal growth in the inflamed gut [18], [26]. Tetrathionate and nitrate may be a limiting resource, requiring *S.* Typhimurium to actively seek out niches containing these electron acceptors. Here we show that energy taxis enhances the fitness of *S.* Typhimurium in the inflamed intestine, by enabling the pathogen to migrate towards favorable metabolic niches (Fig. S6).

While Aer and Tsr, both mediate energy taxis, our data suggest that the functions of these MCPs were not redundant *in vivo*. To migrate towards an anaerobic niche containing tetrathionate by Aer-mediated energy taxis, bacteria need to transfer electrons from NADH/H+ through the quinone pool onto the terminal electron acceptor tetrathionate (tetrathionate respiration), thereby altering the redox state of the cell. The flavin cofactor bound by Aer is proposed to sense changes in the redox state by becoming oxidized or reduced through interaction with a component of the electron transport chain, a process that generates the on and off signal for Aer-mediated energy taxis [21], [26]. Due to this mechanism, the ability to respire tetrathionate was essential for migrating towards higher concentrations of tetrathionate by energy taxis. *S.* Typhimurium strains that were unable to respire tetrathionate neither exhibited Aer-mediated energy taxis towards tetrathionate *in vitro* (Fig. 2) nor benefited from Aer-mediated energy taxis *in vivo* (Fig. 1). Collectively, these data implied that Aer mediates energy taxis towards spatial niches containing tetrathionate in the inflamed intestine.

In contrast, the competitive growth benefit conferred by Tsr did not require *S.* Typhimurium to respire tetrathionate (Fig. 1), indicating that Tsr does not mediate energy taxis towards this electron acceptor. Respiration of 1.3 mol of tetrathionate yields 1 mol of ATP [29], suggesting that tetrathionate respiration is not a strong generator of proton-motive force. This might explain why the proton-motive force sensor Tsr [20] did not mediate energy taxis towards tetrathionate. The nitrate/nitrite redox couple has a much higher standard redox potential (E° = 433 mV) [30] than the tetrathionate/thiosulfate redox couple (E° = 170 mV) [31] and nitrate reductases transport protons by scalar chemistry [24]. The fact that nitrate is a better generator of proton motive force might explain why Tsr sensed nitrate, but not tetrathionate. While Aer mediated energy taxis towards high concentrations of nitrate, only Tsr was functional when concentrations of nitrate were low. Our data show that *in vivo* Tsr-mediated energy taxis was nitrate respiration-dependent, while Aer-mediated energy taxis required tetrathionate respiration. Collectively, these observations suggest that concentrations of nitrate in the gut lumen of mice infected with *S.* Typhimurium are relatively low, thereby rendering Tsr the sole energy taxis sensor of nitrate *in vivo*.

Homologues of Aer have been characterized in *E. coli* [21], *Vibrio cholerae* [32], *P. aeruginosa* [33] and *Campylobacter jejuni* [34]. It has been proposed that energy taxis might be important in the upper gastrointestinal tract [22] where oxygen is a limiting parameter for growth [35]. Our work is the first to suggest that in the increasingly anaerobic environments of the terminal ileum and large bowel, energy taxis becomes important during conditions of intestinal inflammation, because ROS and RNS species generated during this response produce more favorable spatial niches containing respiratory electron acceptors. The ability to use energy taxis to migrate towards such favorable niches confers a growth benefit upon *S*. Typhimurium, and likely upon other facultative anaerobic bacteria.

It has been shown recently that nitrate generated as a by-product of the host inflammatory response supports a bloom of commensal *E. coli* by anaerobic respiration [28]. The picture emerging from this and previous studies [18], [26], [36] is that anaerobic respiration is one of the fundamental principles that boost growth of both commensal and pathogenic Enterobacteriaceae over the obligate anaerobic Clostridia and Bacteroidia during inflammation. The resulting outgrowth could lead to a competition between commensal Enterobacteriaceae and *S*. Typhimurium over nutrients, provided both microbes are present in the same animal. One factor important during the ensuing battle might be antimicrobial proteins, such as lipocalin-2. Lipocalin-2 is released into the intestinal lumen during inflammation [10] and prevents bacterial iron acquisition by binding enterobactin, a siderophore commonly produced by commensal Enterobacteriaceae [37], [38], [39]. The *iroN iroBCDE* gene cluster of *S. enterica* confers resistance to the antimicrobial protein lipocalin-2 and provides a growth advantage in the inflamed gut [10]. Lipocalin-2 resistance might confer an advantage upon *S. enterica* during its competition with commensal Enterobacteriaceae, provided that the latter rely on enterobactin for iron acquisition. However, additional work is needed to elucidate the mechanisms used by *S*. Typhimurium to compete with commensal Enterobacteriaceae in the lumen of the inflamed gut.

## Materials and Methods

### Bacterial strains and growth conditions

The bacterial strains used in this study are listed in Table 1. Unless indicated otherwise, bacteria were grown aerobically at 37°C in LB broth (10 g/l tryptone, 5 g/l yeast extract, 10 g/l NaCl) or on LB agar plates (15 g/l agar). Antibiotics were added to the media at the following concentrations: 0.03 mg/ml chloramphenicol (Cm), 0.1 mg/ml carbenicillin (Carb), 0.05 mg/ml kanamycin (Kan), 0.05 mg/ml nalidixic acid (Nal), and 0.01 mg/ml tetracycline (Tet). When appropriate, the chromogenic substrate of the acidic phosphatase PhoN (5-Bromo-4-chloro-3-indolyl phosphate) was added to solid media at a concentration of 40 mg/l.

**Table 1:** Bacterial strains used in this study.

Strain	Genotype	Source/Reference
*S*. Typhimurium strains		
ATCC 14028	Wild-type isolate of *S*. *enterica* serotype Typhimurium	ATCC
IR715	Nalidixic acid-resistant derivative of ATCC 14028	[44]
AJB715	IR715 Δ*phoN*::Kan^R^	[45]
AT350	*cheY*::Tn*10* (Tet^R^)	[46]
FR4	IR715 *tsr*::pFR3 (Cm^R^)	This Study
FR5	IR715 *aer*::pFR2 (Carb^R^)	This Study
FR6	IR715 *aer*::pFR2 *tsr*::pFR3 (Carb^R^, Cm^R^)	This Study
FR7	IR715 *aer*::pFR4 (Cm^R^)	This Study
FR8	IR715 *ttrA*::pSW171 *tsr*::pFR3 (Carb^R^, Cm^R^)	This Study
FR9	IR715 *ttrA*::pSW171 *aer*::pFR4 (Carb^R^, Cm^R^)	This Study
FR10	IR715 Δ*invA*::*tetRA* Δ*spiB*::KSAC *tsr*::pFR3 (Tet^R^, Kan^R^, Cm^R^)	This Study
FR11	IR715 Δ*invA*::*tetRA* Δ*spiB*::KSAC *aer*::pFR2 (Tet^R^, Kan^R^, Carb^R^)	This Study
FR13	IR715 *cheY*::Tn*10* (Tet^R^)	This Study
FR35	IR715 *mcpC*::Cm^R^	This Study
FR36	IR715 *mcpB*::Kan^R^	This Study
FR37	IR715 *mcpA*::Kan^R^	This Study
FR38	IR715 Δ*aer*	This Study
FR42	IR715 *trg*::Cm^R^	This Study
FR43	IR715 Δ*invA* Δ*spiB trg*::Cm^R^	This Study
FR46	IR715 *tsr*::pFR3 (Cm^R^) Δ*napA* Δ*narZ narG*::pCAL5	This Study
FR47	IR715 *aer_S22F_*	This Study
CAL50	IR715 Δ*napA* Δ*narZ narG*::pCAL5	[26]
MB193	14028 *mcpA*::Kan^R^	This Study
QW111	14028 *mcpB*::Kan^R^	This Study
SM15	14028 *trg*::Cm^R^	This Study
SM19	14028 *mcpC*::Cm^R^	This Study
SPN452	IR715 Δ*invA*::*tetRA* Δ*spiB*::KSAC (Tet^R^; Kan^R^)	[10]
SPN453	IR715 Δ*invA*::KSAC (Kan^R^)	This Study
SPN455	IR715 Δ*invA* (unmarked deletion)	This Study
SPN458	IR715 Δ*spiB*::pSPN56(Cm^R^, Tet^R^, Δ*spiB*)	[30]
SPN487	IR715 Δ*invA* Δ*spiB* (unmarked deletions)	This Study
SW215	IR715 *flgK5396*::Mu*d*J	[47]
SW661	IR715 *ttrA*::pSW171 (Carb^R^)	[18]
*E. coli* strains		
DH5α λ*pir*	F^−^ *endA1 hsdR17* (r^−^ m^+^) *supE44 thi-1 recA1 gyrA96 relA1* Δ(*lacZYA*-*argF*)U169 *deoR nupG* Φ80*lacZ*ΔM15 λ*pir*	[48]
S17-1 λ*pir*	*zxx*::RP4-2-Tet^r^::Mu-Kan^r^::Tn*7 recA1 thi pro hsdR* (r^−^ m^+^) λ*pir*	[49]
TOP10	F^−^ *mcrA* Δ(*mrr*-*hsdRMS*-*mcrBC*) Φ80*lacZ*ΔM15 *lacX74 recA1 araD139* Δ(*ara - leu*)*7697 galE15 galK rpsL endA1 nupG*	Invitrogen

* Cm^R^: chloramphenicol resistance; Carb^R^: carbenicillin resistance; Kan^R^: kanamycin resistance, Tet^R^: tetracycline resistance.

To determine competitive indices (CI) after *in vitro* growth, LB broth or LB broth containing either 10 mM tetrathionate or 10 mM nitrate was inoculated with an equal mixture of two bacterial strains (at a density corresponding to a 1∶200 dilution of an overnight culture) and grown anaerobically at 37°C (Bactron I anaerobic chamber; Sheldon Manufacturing) overnight. The cultures were spread on agar plates containing the appropriate antibiotics to determine the ratio of recovered bacteria. Three technical replicates were performed for each experiment and each experiment was repeated at least three times independently.

### Construction of plasmids

The plasmids and primers used in this study are listed in Tables 2 and 3, respectively. PCR products were routinely cloned into pCR2.1 using the TOPO TA cloning kit (Invitrogen, Carlsbad) and sequenced (SeqWright Fisher Scientific, Houston) prior to subcloning into appropriate vectors.

**Table 2:** Plasmids used in this study.

Designation	Relevant Characteristics	Reference/source
pBS34	pBluescript II KS+ (Carb^R^, Kan^R^, KSAC cassette)	[10]
pCR2.1	Cloning vector; Carb^R^, Kan^R^	Invitrogen
pEP185.2	*oriR6K mobRP4 cat* (Cm^R^)	[50]
pFR2	*‘aer’* gene fragment cloned into pGP704; insertion of pFR2 into the IR715 chromosome *aer* gene.	This Study
pFR3	*‘tsr’* gene fragment cloned into pEP185.2; insertion of pFR3 into the IR715 chromosome disrupts *tsr*.	This Study
pFR4	*‘aer’* gene fragment cloned into pEP185.2; insertion of pFR4 into the IR715 chromosome disrupts *aer*.	This Study
pFR5	*aer open reading frame and promoter cloned into pWSK29*	This Study
pFR6	*tsr open reading frame and promoter cloned into pWSK29*	This Study
pKD3	oriR6K, Cm^R^	[42]
pKD4	oriR6K, Kan^R^	[42]
pKD46	Plasmid encoding lambda red recombinase	[42]
pSPN57	pRDH10(Cm^R^, Δ*invA(−9 to +2057)*)	[10]
pSPN60	pSPN57(Cm^R^, Kan^R^, Δ*invA*::KSAC)	This Study
pWSK29	Cloning vector; Carb^R^	[51]
pWSK129	Cloning vector; Kan^R^	[51]

**Table 3:** Primers used in this study.

Target gene (purpose)/organism	Sequence
*aer* (cloning)/*S*. Typhimurium	5′-GTCGACCGGACTCCGCCATCTGG-3′
	5′-GAGCTCCTATTGGAATGGCAAATTGTGC-3′
*aer (*complementation)/*S*. Typhimurium	5′-TCTAGATTTAGCGTTCGCTTCTCGAT-3′
	5′-GAGCTCCCTTCTATCAGAGCGCATCC-3′
*aer* (RT PCR)/*S*. Typhimurium	5′-CACGGTTCGCCCTGTTTTAG-3′
	5′- CTCACGCCAATGACACTTTTG-3′
*aer* (deletion)/*S*. Typhimurium	del_A 5′-GTCGACTGAATTAAGTATTGCAGGGATTAGC-3′
	del_B 5′-TGACCGCATCAGAAGACATGATAGCGTCCTGTG-3′
	del_C 5′-CATGTCTTCTGATGCGGTCACGGTACTGC-3′
	del_D 5′-GGATCCTTTCACCGTGGTGGGATAAT-3′
*aer* (point mutant)/*S*. Typhimurium	S22F_B 5′-GGTCGTAAACATCAGAGTGGTATC-3′
	S22F_C 5′-GATGTTTACGACCGACCTGG-3′
*gmk* (RT PCR)/*S*. Typhimurium	5′-TTGGCAGGGAGGCGTTT-3′
	5′-GCGCGAAGTGCCGTAGTAAT-3′
*invA* (deletion presence)/*S.* Typhimurium	5′-TTCGTCGTAAAGACCTGG-3′
	5′-GGTAATCAGCGGTTCACG-3′
*invA* (gene presence)/*S.* Typhimurium	5′-CTTTACGGTTCCTTTGACG-3′
	5′-ATTACGCACGGAAACACG-3′
KSAC (outwards cassette primers)/*S.* Typhimurium	5′-GGCATAAATTCCGTCAGC-3′
	5′-TGATGACGAGCGTAATGG-3′
*mcpA* (cloning)/*S*. Typhimurium	5′-AATGTTGTCAAAATACCGCCGCCAGCAGCAGAATCTGGCGTGTAGGCTGGAGCTGCTTC-3′
	5′-TTCCAGCTGGTTAACCACCTCATTACTGGATCGCTCCATATATGAATATCCTCCTTAGT-3′
*mcpB* (cloning)/*S*. Typhimurium	5′-CTAATCTCTTATATTCAGCAAGGAAATTCATATGCGTTTGGTGTAGGCTGGAGCTGCTTC-3′
	5′-GCCAGCGCATTACGCGCTTTTTTGCTTCACATCAGAAGGAACATATGAATATCCTCCTTAG-3′
*mcpC* (cloning)/*S*. Typhimurium	5′-GTTTTTGCATAACATTAAAATACGTTCAAAATTATTTATGGTGTAGGCTGGAGCTGCTTC-3′
	5′-CCTCTTCGCGGACGCGGAACACGTTAACCAGCTCTTTTAACATATGAATATCCTCCTTAG -3′
*mcpC* (RT PCR)/*S*. Typhimurium	5′-AGAGAGTTTGGTCGCTTCAC-3′
	5′-ATGGAACAGATTACCGCCAC-3′
*spiB* (deletion presence)/*S*. Typhimurium	5′-GGGTCGTTAGTGTATTGC-3′
	5′-CTGTTGACTCACCTTAGC-3′
*spiB* (gene presence)/*S.* Typhimurium	5′-TGGCTGAATGAAGGTAACC-3′
	5′-CTCAGATGGACAATTTCTCC-3′
*trg* (cloning)/*S*. Typhimurium	5′-TATTCGGCTGGTTCCGCTGTTTTCCTCCATTCTCGGTGGCGTGTAGGCTGGAGCTGCTTC-3′
	5′-AGCGGGCAGCCTGTTCTTCAAGCGAACGTGCCGCCGCGGACATATGAATATCCTCCTTAG-3′
*tsr* (cloning)/*S*. Typhimurium	5′-CGTCGACTACGATATTGCTGTTGAGG-3′
	5′-GAGCTCATCGTTGTTGCCCATCG-3′
*Gapdh* (RT PCR)/*Mus musculus*	5′-TGTAGACCATGTAGTTGAGGTCA-3′
	5′-AGGTCGGTGTGAACGGATTTG-3′
*Kc* (RT PCR)/*Mus musculus*	5′-TGCACCCAAACCGAAGTCAT-3′
	5′-TTGTCAGAAGCCAGCGTTCAC-3′
*Nos2* (RT PCR)/*Mus musculus*	5′-CCAGCCTTGCATCCTCATTGG-3′
	5′-CCAAACACCAAGCTCATGCGG-3′

RT PCR, quantitative real-time PCR.

An internal fragment of the *aer* gene was amplified by PCR using the primers listed in Table 3. The *aer* PCR product was then cloned into the suicide plasmids pGP704 and pEP185.2 using the SalI and SacI restriction enzyme sites to construct pFR2 and pFR4, respectively. A fragment of the *tsr* gene was amplified by PCR and subcloned into pEP185.2 using the SalI and SacI restriction enzyme sites to create pFR3. The KSAC kanamycin resistance cassette of pBS34 was subcloned into the XbaI restriction site of pSPN57, generating a Kan-marked allelic exchange *invA* deletion construct (pSPN60).

A suicide plasmid for generating a deletion of the *aer* gene was constructed by using primers del_A and del_B to PCR amplify a 404 bp 5′ flanking sequence that included 9 nucleotides of the *aer* coding region. Primers del_C and del_D were used to PCR amplify a 573 bp fragment that included 24 nucleotides of the 3′ coding region. The two PCR fragments were joined using the splicing overlap extension (SOE) technique [40], [41] and the resulting PCR product containing an in-frame *aer* deletion was digested with BamHI and SalI and cloned into pRDH10 to generate plasmid pFR7.

A suicide plasmid for introducing a point mutation into the *aer* open reading frame was constructed by PCR amplifying a 5′ fragment of the *aer* gene using primer del_A, which anneals 395 bps upstream of the gene and primer S22F_B, which contains a missense mutation (C65T). Primer S22F_C, which encodes the same missense mutation and primer del_D were used to amplify the remaining *aer* ORF plus 549 bps downstream of the *aer* stop codon. The two PCR fragments were joined using the SOE technique [40], [41] and the resulting PCR product containing an *aer* allele with a C65T substitution was digested with BamHI and SalI and cloned into pRDH10 to generate pFR8.

For complementation of the *aer* mutant, the *aer* open reading frame including the promoter region was amplified using the primers listed in Table 3. The resulting PCR fragment was cloned into pWSK29 using XbaI and SacI to create pFR5.

For complementation of the *tsr* mutant, the *tsr* open reading frame including the promoter region was amplified using the primers listed in Table 3. The resulting PCR fragment was cloned into pWSK29 using XbaI and SacI to create pFR6.

### Construction of *S*. *Typhimurium* mutants by allelic exchange

Suicide plasmids were propagated in *E. coli* DH5α λ*pir* and introduced into the *S*. Typhimurium strain IR715 by conjugation using *E. coli* S17-1 λ*pir* as a donor strain. Transconjugants were selected on LB plates containing nalidixic acid (IR715) and antibiotics selecting for integration of the suicide plasmid. Integration of the suicide plasmids was verified by PCR. Using this methodology, pFR2, pFR4 and pFR3 were integrated into the *aer* and *tsr* genes of IR715 to yield strains FR5, FR7 and FR4, respectively. An *mcpA* mutant (MB193) *mcpB* mutant (QW111), *mcpC* mutant (SM19) and *trg* mutant (SM15) were constructed by the one-step mutagenesis method [42]. Primers were designed with overhang sequences complementarily to the beginning and end of the Cm cassette in pKD3 to replace most of the *mcpC* gene or the *trg* gene with a Cm cassette, generating SM19 and SM15, respectively. Primers were designed with overhang sequences complementarily to the beginning and end of Kan cassette in pKD4 to replace most of the *mcpA* or *mcpB* open reading frames with a Kan cassette to generate strains MB193 and QW111, respectively.

The *invA* locus of IR715 was deleted with the KSAC Kan resistance cassette by conjugating pSPN60 from S17-1 λ*pir* into IR715, selecting for double-crossover transconjugants on LB plates containing Nal and Kan. A strain positive for amplification with primers directed outwards from KSAC and primers directed inwards from beyond the flanking regions employed in the deletion of *invA* was labeled SPN453. An unmarked *invA* deletion mutant was then generated by conjugating pSPN57 from S17-1 λ*pir* into SPN453 by following a previously described methodology [30]. A strain positive by PCR for the unmarked *invA* deletion and negative for *invA* amplification was termed SPN455.

The *aer* was deleted by conjugation of pFR7 from *E. coli* S17-1 λ*pir* into *S.* Typhimurium strain IR715. Transconjugants were selected for on LB plates containing Nal and Cm. Sucrose counter-selection was performed as described previously [43]. A strain that was sucrose-tolerant and Cm^s^ was verified by PCR to carry a deletion in *aer* and was denoted FR38.

The *aer*_S22F_ mutation was constructed by conjugation of pFR8 from *E. coli* S17-1 λ*pir* into *S.* Typhimurium strain FR38. Transconjugants were selected for on LB plates containing Nal and Cm. Sucrose counter-selection was performed as described previously [43]. A strain that was sucrose-tolerant and Cm^s^, denoted FR47, was verified by PCR amplification and sequence analysis.

### Generalized phage transduction

Phage P22 HT105/1 *int-201* was used for generalized transduction. Transductants were routinely purified from phage contamination on Evans blue-Uranine agar and then cross-struck against P22 H5 to confirm phage sensitivity. The *ttrA::*pSW171 mutation from SW661 was introduced into FR4 and FR7 to yield strains FR8 and FR9, respectively. The *tsr*::pFR3 or *aer*::pFR2 mutations were introduced into the SPN452 to create strains FR10 and FR11, respectively. The *cheY*::Tn*10* mutation from AT350 was transduced into IR715 to generate strain FR13. The *mcpC*::Cm mutation from SM19 was transduced into IR715 to generate FR35. The *mcpB*::Kan mutation from QW111 was transduced into IR715 to generate FR36. The *mcpA*::Kan mutation from MB193 was transduced into IR715 to generate FR37.The *trg*::Cm mutation from SM15 was transduced into IR715 to generate FR42.

An unmarked *spiB* deletion was generated in SPN455 by transducing the merodiploid Δ*spiB*::pSPN56(Δ*spiB*) locus from SPN458 followed by sucrose selection as previously described [30]. A strain positive by PCR for the unmarked *spiB* deletion and negative for *spiB* amplification was dubbed SPN487. The *trg*::Cm mutation of SM15 was then transduced into SPN487, creating FR43.

### Animal experiments

Mouse and calf experiments adhered to USDA guidelines and were approved by the Institutional Animal Care and Use Committees at the University of California at Davis and Texas A&M University, respectively.

#### Mouse colitis model

Female C57BL/6J mice, aged 9–12 weeks, were obtained from The Jackson Laboratory (Bar Harbor). Animals were pretreated with 20 mg Streptomycin and orally inoculated 24 hours later with 0.1 ml LB broth or *S*. Typhimurium in 0.1 ml LB broth as described previously [11]. For competitive infections, mice were inoculated with an equal mixture of 5×10^8^ CFU of each strain. For single strain infections, animals were inoculated with approximately 1×10^9^ CFU per animal. Animals were euthanized 4 days after infection and organs were collected for RNA purification and bacteriological analysis as described previously [18]. For competitive infections, the ratio of recovered wild-type bacteria to mutant bacteria (output ratio) was divided by the ratio present in the inoculum (input ratio) to determine the competitive index (CI).

#### Mouse typhoid model

Female CBA/J mice, aged 6–8 weeks, were obtained from The Jackson Laboratory (Bar Harbor). Animals were inoculated with 0.1 ml of a suspension (LB broth) containing an equal mixture of 5×10^8^ CFU of each bacterial strain. Animals were euthanized at the indicated time points to collect organs.

#### Bovine ligated ileal loop model

Bovine ligated ileal loops surgery was performed as described previously [27]. Briefly, bovine loops were infected by intralumenal injection of 3 ml of a suspension of bacterial strains in LB broth containing an equal mixture of 5×10^8^ CFU of each strain as indicated. 8 h after infection, loops were resected and luminal contents collected. Loops were opened longitudinally and 6 mm tissue biopsy punches were collected, and the homogenized tissue spread on selective media. Each bacterial strain was tested in at least three different animals. To facilitate recovery of *Salmonella* strains from biological samples, the wild-type strain (IR715) was marked with a mutation in the *phoN* gene (AJB715).

### Motility plate assay

Soft agar motility plates containing LB and 0.3% (w/v) agar or soft agar plates supplemented with 5 mM tetrathionate were inoculated with single colonies and incubated at 37°C anaerobically (GasPak system, BD Biosciences) for 6.5 hours and the area of the halo was determined (Labworks 4.6 software, UVP). Each experiment was performed in triplicate.

### Capillary assay

Capillary assays were performed as described previously [23] with the modifications indicated below. An overnight culture was diluted 1∶100 in LB broth and grown anaerobically at 37°C (Bactron I anaerobic chamber; Sheldon Manufacturing) to late exponential phase (OD_600_ between 0.6–0.8). An equal volume of each strain was added to a tube and washed twice (3,220 g, 5 minutes) in 0.5 ml of chemotaxis buffer (CB; 10 mM potassium phosphate buffer (pH 7.4), 0.1 mM EDTA). The pellet was resuspended in CB to a concentration of between 1×10^7^ and 1×10^8^ CFU/ml and 100 µl of the culture was added to a 1.5 ml tube. To make the capillaries, 1 µl glass capillaries that had been sealed on one end were briefly flamed and then added to 1.5 ml microcentrifuge tubes containing tetrathionate or nitrate at the indicated concentrations. The glass capillary was then placed into a 1.5 ml microcentrifuge tube containing *S*. Typhimurium strains and incubated at 37°C inside the anaerobe chamber for 45 minutes. The glass capillary was removed, and wiped off and dilutions of the contents of the capillary tube were spread on agar plates containing the appropriate antibiotics. Three technical replicates were performed for each experiment and each experiment was repeated at least three times independently.

### RT-PCR and real time PCR analysis

Eukaryotic gene expression was determined by real-time PCR as previously described [18]. Briefly, eukaryotic RNA was isolated using TRI reagent (Molecular Research Center, Cincinnati). A Reverse transcriptase reaction was performed to prepare complementary DNA (cDNA) using TaqMan reverse transcription reagents (Applied Biosystems, Carlsbad). A volume of 4 µl of cDNA was used as template for each real-time PCR reaction in a total reaction volume of 25 µl. Real-time PCR was performed using SYBR-Green (Applied Biosystems) along with the primers listed in Table 3. Data were analyzed using the comparative Ct method (Applied Biosystems). Transcript levels of *Nos2* and *Kc* were normalized to mRNA levels of the housekeeping gene *Gapdh*.

#### Bacterial gene expression

Bacterial RNA was isolated using the Aurum Total RNA Kit (Bio-Rad) according to the recommendations of the manufacturer. Afterward, an additional DNase treatment was performed using the Ambion DNA removal and inactivation kit. RT-PCR and real-time PCR were performed as described previously [36]. Transcription of the *mcpC* in each sample was normalized to the respective levels of guanyl nucleotide kinase mRNA, encoded by the *gmk* gene.

### Histopathology

Formalin fixed cecal tissue sections were stained with hematoxylin and eosin, and a veterinary pathologist performed a blinded evaluation using criteria published previously [36]. Representative images were obtained using an Olympus BX41 microscope and the brightness adjusted (Adobe Photoshop CS2).

### Statistical analysis

Fold changes of ratios (bacterial numbers or mRNA levels) were transformed logarithmically prior to statistical analysis. An unpaired Student's *t*-test was used to determine whether differences in fold changes between groups were statistically significant (P<0.05). Significance of differences in histopathology scores was determined by a one-tailed non-parametric test (Mann-Whitney).

## Supporting Information

Figure S1 **Inflammatory changes in the cecum in the mouse colitis model.** For selected experiments shown in Figure 1, blinded histopathology scoring was performed. The graph shows averages (lines) of combined blinded histopathology scores for individual animals (filled circles).
(PDF)

Figure S2 **Polarity of** ***aer*** **::pFR4 on expression of** ***mcpC*** **.** (A) Schematic representation of the genetic region surrounding the *aer* gene. (B) Expression levels of *mcpC* were determined by quantitative real-time PCR with primers listed in Table 3. Each experiment was repeated three times independently. Data represent geometric means ± standard error of mRNA levels detected for *aer* (white bar) relative to mRNA levels in the *S.* Typhimurium wild-type (wt) strain (AJB715), which were set to 100%.
(PDF)

Figure S3 **Anaerobic growth of** ***S.*** **Typhimurium strains on motility plates and in LB broth.** (A) Halo size (arbitrary units) around a point of inoculation determined after 6.5 hours anaerobic incubation in the motility plate assay. Bars represent averages from three independent experiments ± standard error. *, *P*<0.05; ns, not significantly different; nh, no halo. (B) Representative images of halos produced in the motility plate assay. (C) Competitive growth of the indicated mixtures of *S.* Typhimurium strains in LB broth under anaerobic conditions in the absence (−) or presence (+) of tetrathionate (S_4_O_6_^2−^, 10 mM) or nitrate (NO_3_^−^, 10 mM). Bars represent geometric means of competitive indices (CI) from at least three independent experiments ± standard error.
(PDF)

Figure S4 **Aer boosts growth in the mouse typhoid model.** Groups (*N = 6*) of genetically resistant CBA mice were inoculated with the *S*. Typhimurium wild-type strain (wt) and an *aer* mutant (*aer*) or with a *ttrA* mutant (*ttrA*) and a *ttrA aer* mutant (*ttrA aer*) and organs were collected for analysis on the indicated days after infection. Bars represent geometric means of competitive indices (CI) recovered from colon contents ± standard error. *, *P*<0.05; NR, no bacteria recovered.
(PDF)

Figure S5 **Analysis of histopathological lesions induced by infection with different** ***S.*** **Typhimurium strains.** Groups of streptomycin pre-treated mice (*N* = is indicated in panel A) were inoculated with sterile medium (mock-infected) or with the *S*. Typhimurium wild-type strain (wt), an *aer* mutant (*aer*) or a *cheY* mutant (*cheY*) and organs were collected for analysis four days after infection. (A) Blinded histopathology scoring of cecal inflammation showing averages (bars) of scores for individual animals (circles). (B) Representative images of histopathological changes. (C) Expression of pro-inflammatory markers in the cecal mucosa was determined by quantitative real-time PCR analysis. Bars represent geometric means of *Kc* and *Nos2* mRNA copy numbers as fold-change over mRNA levels in mock-infected mice ± standard error.
(PDF)

Figure S6 **Model for the mechanism by which energy taxis confers a fitness advantage during colitis.** Upon ingestion, a fraction of the luminal *S.* Typhimurium population migrates along a concentration gradient of galactose residues towards the mucus layer [6], but in the absence of inflammation, growth of this population is limited. Another fraction of the population uses its virulence factors to invade the intestinal epithelium (T3SS-1) and survive in macrophages (T3SS-2), which results in intestinal inflammation. Neutrophils recruited during this process transmigrate into the intestinal lumen and produce reactive oxygen species (ROS) and reactive nitrogen species (RNS) to kill bacteria. A by-product of releasing ROS and RNS is the generation of respiratory electron acceptors (tetrathionate and nitrate). The luminal fraction of the *S.* Typhimurium population now uses energy taxis to migrate towards environments containing tetrathionate or nitrate and subsequently uses anaerobic respiration to gain a fitness advantage over competing microbes that grow by fermentation.
(PDF)

Table S1 **Calculation of competitive indices for experiments using the capillary assay.** S, strain number; I, inoculum; G.M, geometric mean; C/ml, colony forming units per ml; CFU/cap, geometric mean of colony forming units per capillary after incubation; IR, input ratio; OR, output ratio; CI, competitive indices; NR, *napA narZ narG* mutant; T (5 mM), capillary containing 5 mM tetrathionate; B, capillary containing buffer; N (1 mM), capillary containing 1 mM nitrate; (O2), experiment was performed under aerobic conditions.
(PDF)
