# Supplementary figures and images for: Salmonella Uses Energy Taxis to Benefit from Intestinal Inflammation
Source: PLoS Pathog. 2013 Apr 18;9(4):e1003267. doi: 10.1371/journal.ppat.1003267 (PMC3630101; doi:10.1371/journal.ppat.1003267)

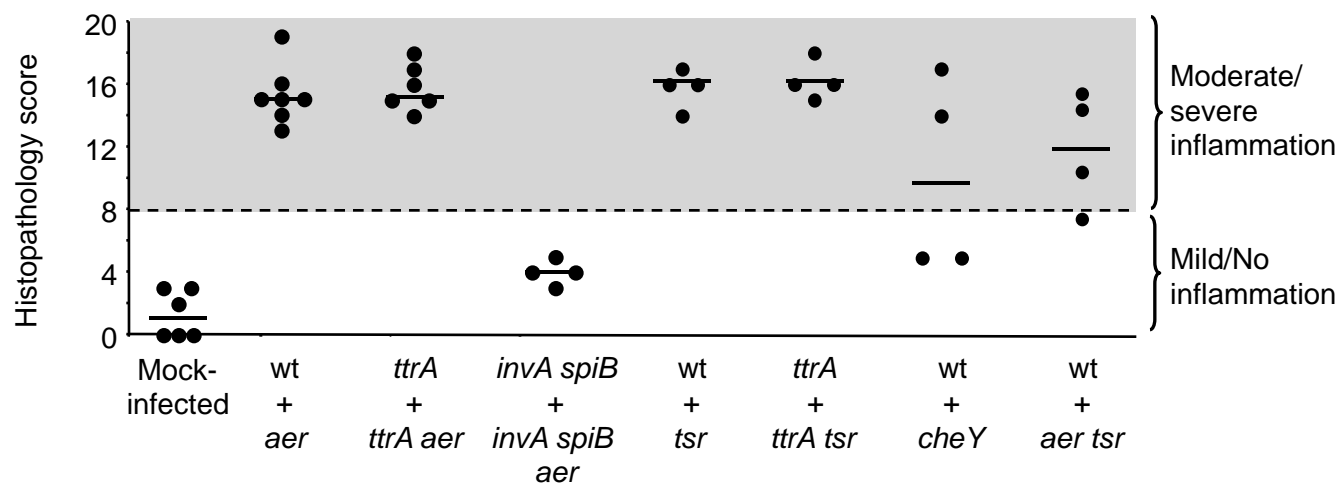

Supplement: Figure S1 — Inflammatory changes in the cecum in the mouse colitis model. For selected experiments shown in Figure 1, blinded histopathology scoring was performed. The graph shows averages (lines) of combined blinded histopathology scores for individual animals (filled circles). (PDF) [file ppat.1003267.s001.pdf]

A

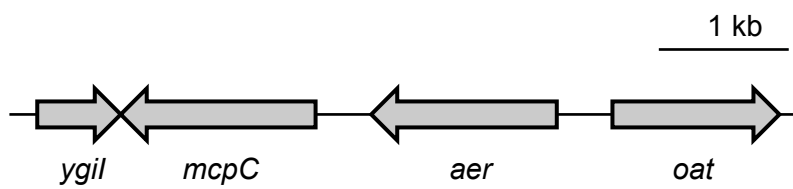

B

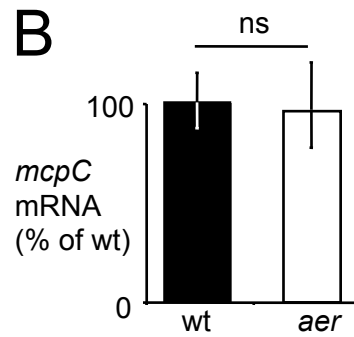

Supplement: Figure S2 — Polarity of aer ::pFR4 on expression of mcpC . (A) Schematic representation of the genetic region surrounding the aer gene. (B) Expression levels of mcpC were determined by quantitative real-time PCR with primers listed in Table 3. Each experiment was repeated three times independently. Data represent geometric means ± standard error of mRNA levels detected for aer (white bar) relative to mRNA levels in the S. Typhimurium wild-type (wt) strain (AJB715), which were set to 100%. (PDF) [file ppat.1003267.s002.pdf]

A

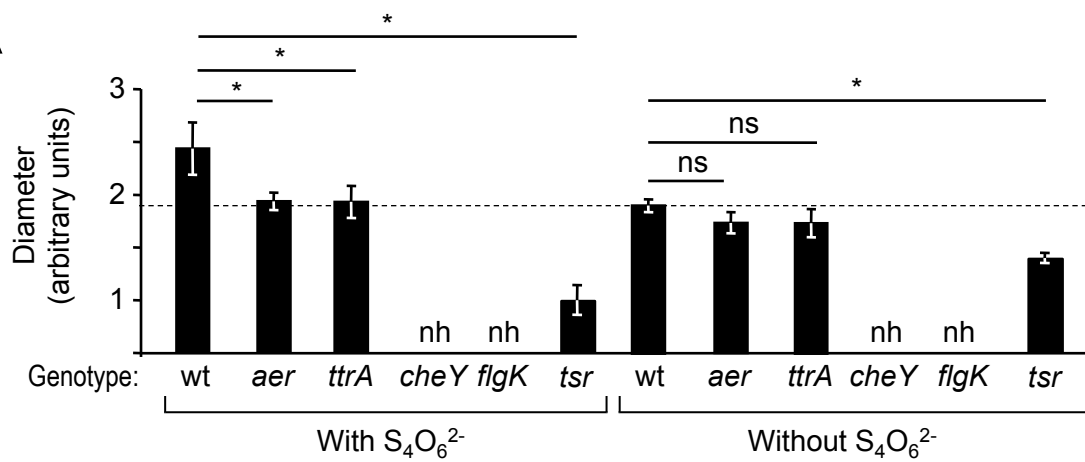

B

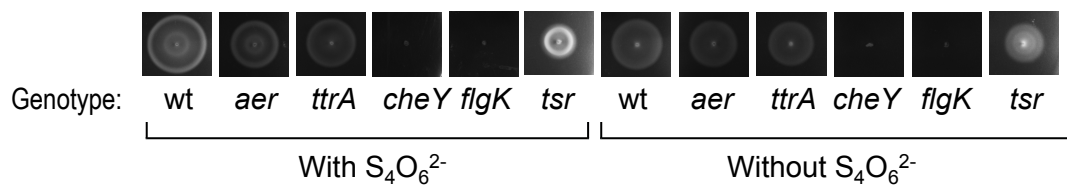

C

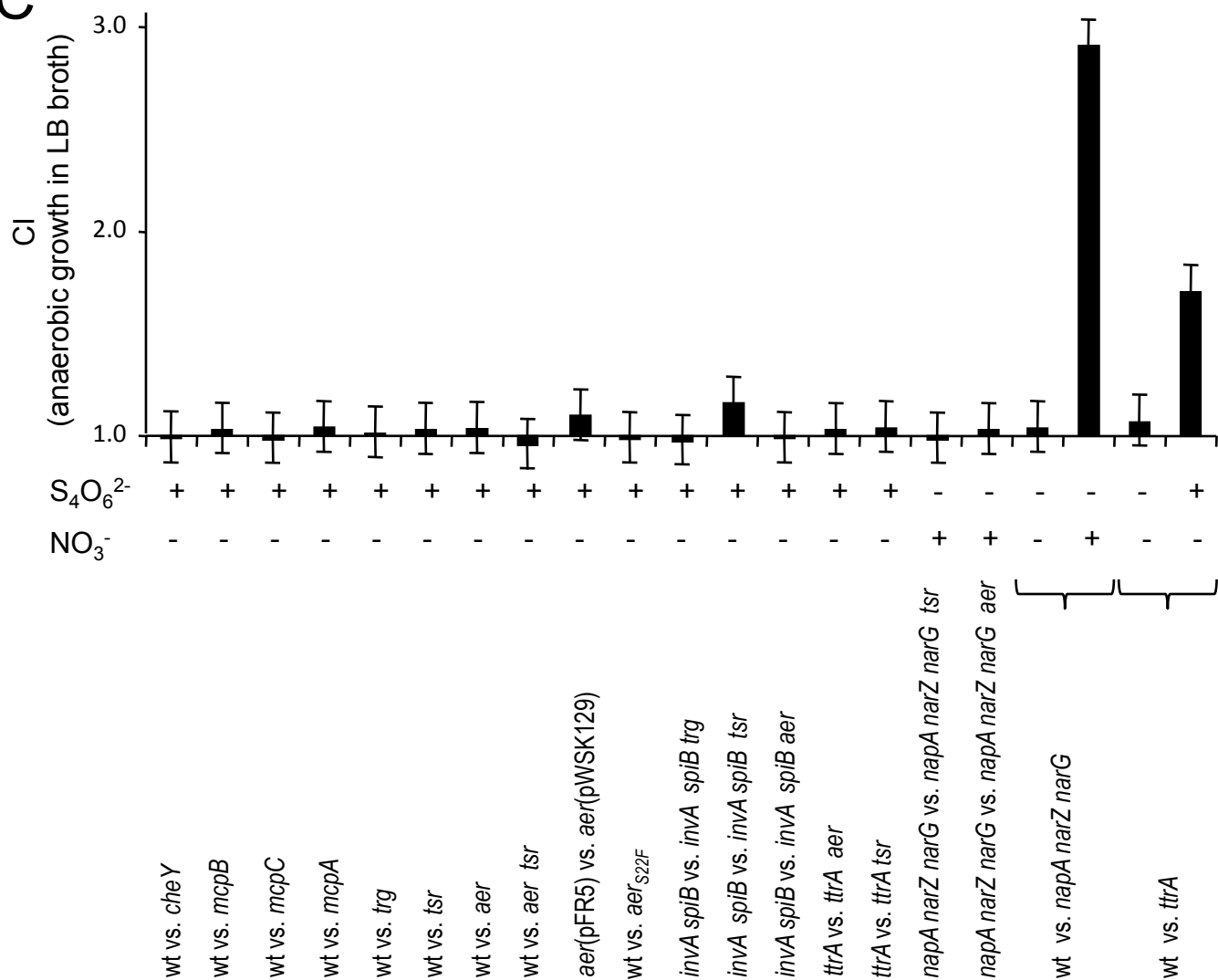

Supplement: Figure S3 — Anaerobic growth of S. Typhimurium strains on motility plates and in LB broth. (A) Halo size (arbitrary units) around a point of inoculation determined after 6.5 hours anaerobic incubation in the motility plate assay. Bars represent averages from three independent experiments ± standard error. *, P<0.05; ns, not significantly different; nh, no halo. (B) Representative images of halos produced in the motility plate assay. (C) Competitive growth of the indicated mixtures of S. Typhimurium strains in LB broth under anaerobic conditions in the absence (−) or presence (+) of tetrathionate (S4O6 2−, 10 mM) or nitrate (NO3 −, 10 mM). Bars represent geometric means of competitive indices (CI) from at least three independent experiments ± standard error. (PDF) [file ppat.1003267.s003.pdf]

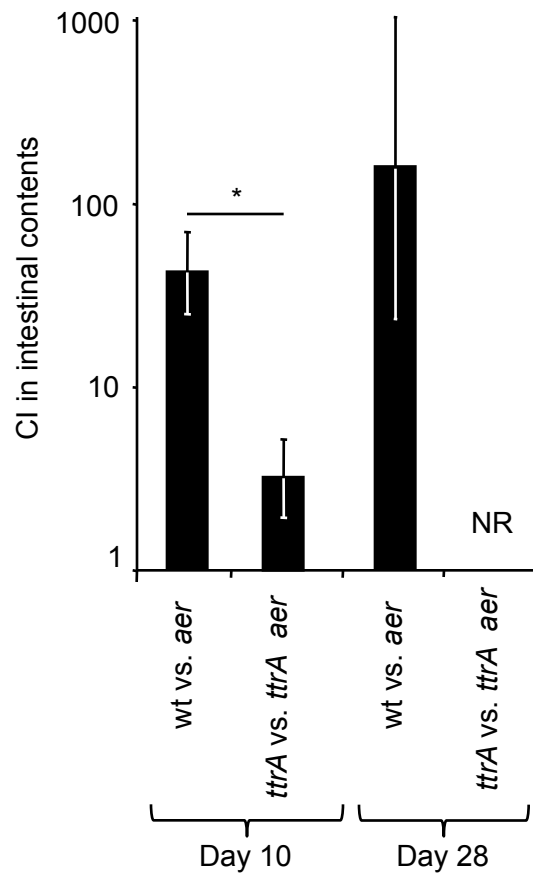

Supplement: Figure S4 — Aer boosts growth in the mouse typhoid model. Groups (N = 6) of genetically resistant CBA mice were inoculated with the S. Typhimurium wild-type strain (wt) and an aer mutant (aer) or with a ttrA mutant (ttrA) and a ttrA aer mutant (ttrA aer) and organs were collected for analysis on the indicated days after infection. Bars represent geometric means of competitive indices (CI) recovered from colon contents ± standard error. *, P<0.05; NR, no bacteria recovered. (PDF) [file ppat.1003267.s004.pdf]

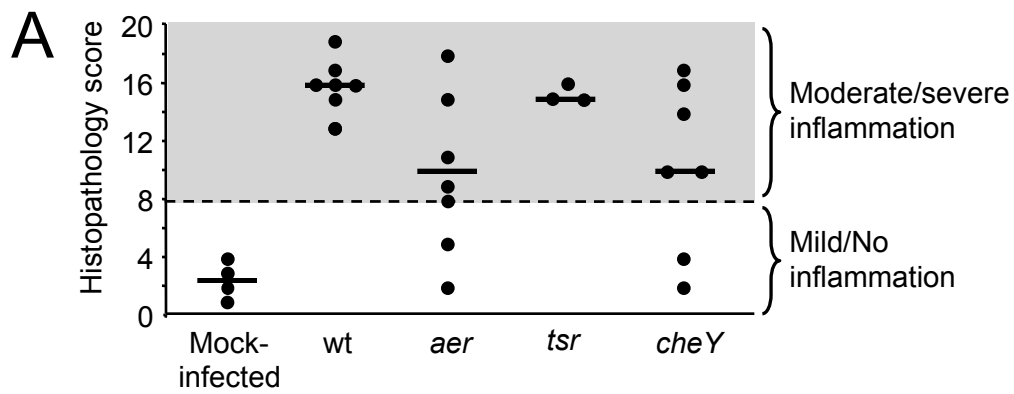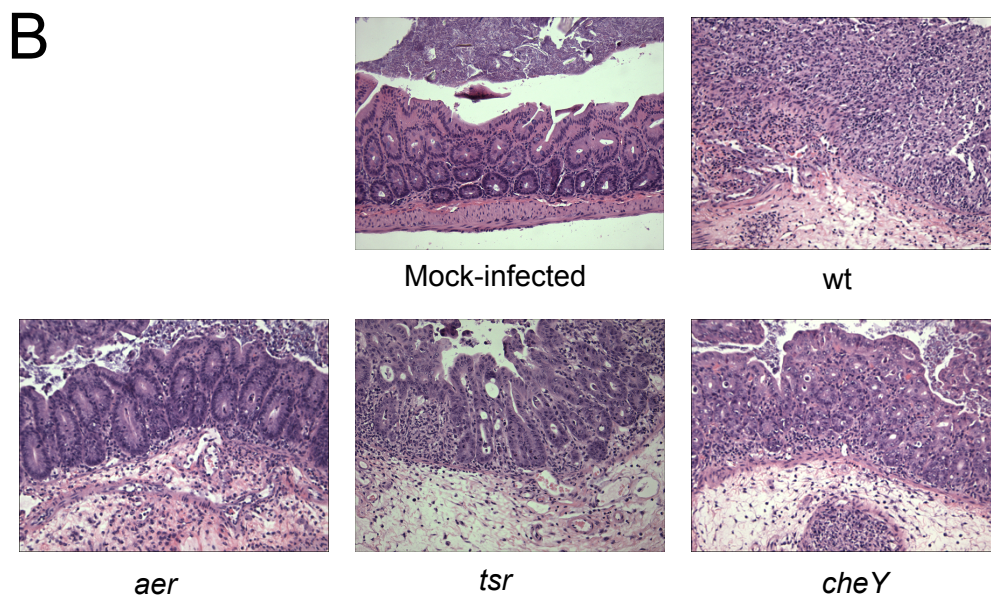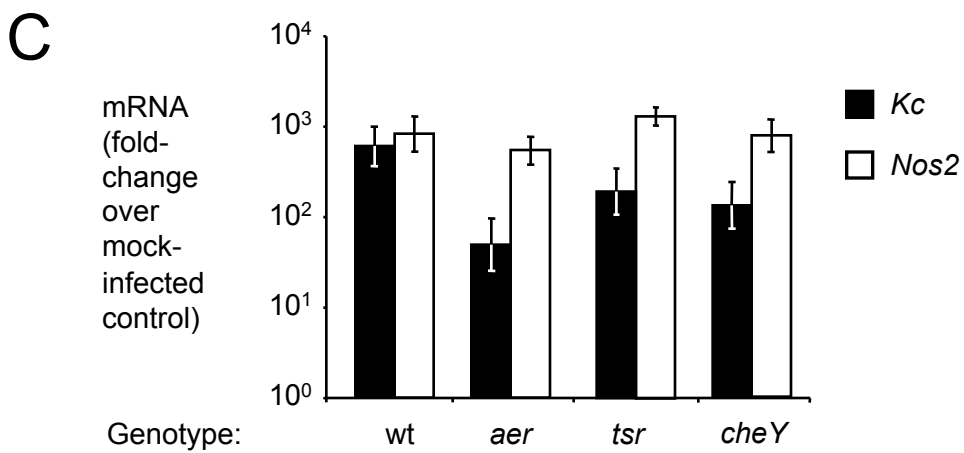

Supplement: Figure S5 — Analysis of histopathological lesions induced by infection with different S. Typhimurium strains. Groups of streptomycin pre-treated mice (N = is indicated in panel A) were inoculated with sterile medium (mock-infected) or with the S. Typhimurium wild-type strain (wt), an aer mutant (aer) or a cheY mutant (cheY) and organs were collected for analysis four days after infection. (A) Blinded histopathology scoring of cecal inflammation showing averages (bars) of scores for individual animals (circles). (B) Representative images of histopathological changes. (C) Expression of pro-inflammatory markers in the cecal mucosa was determined by quantitative real-time PCR analysis. Bars represent geometric means of Kc and Nos2 mRNA copy numbers as fold-change over mRNA levels in mock-infected mice ± standard error. (PDF) [file ppat.1003267.s005.pdf]

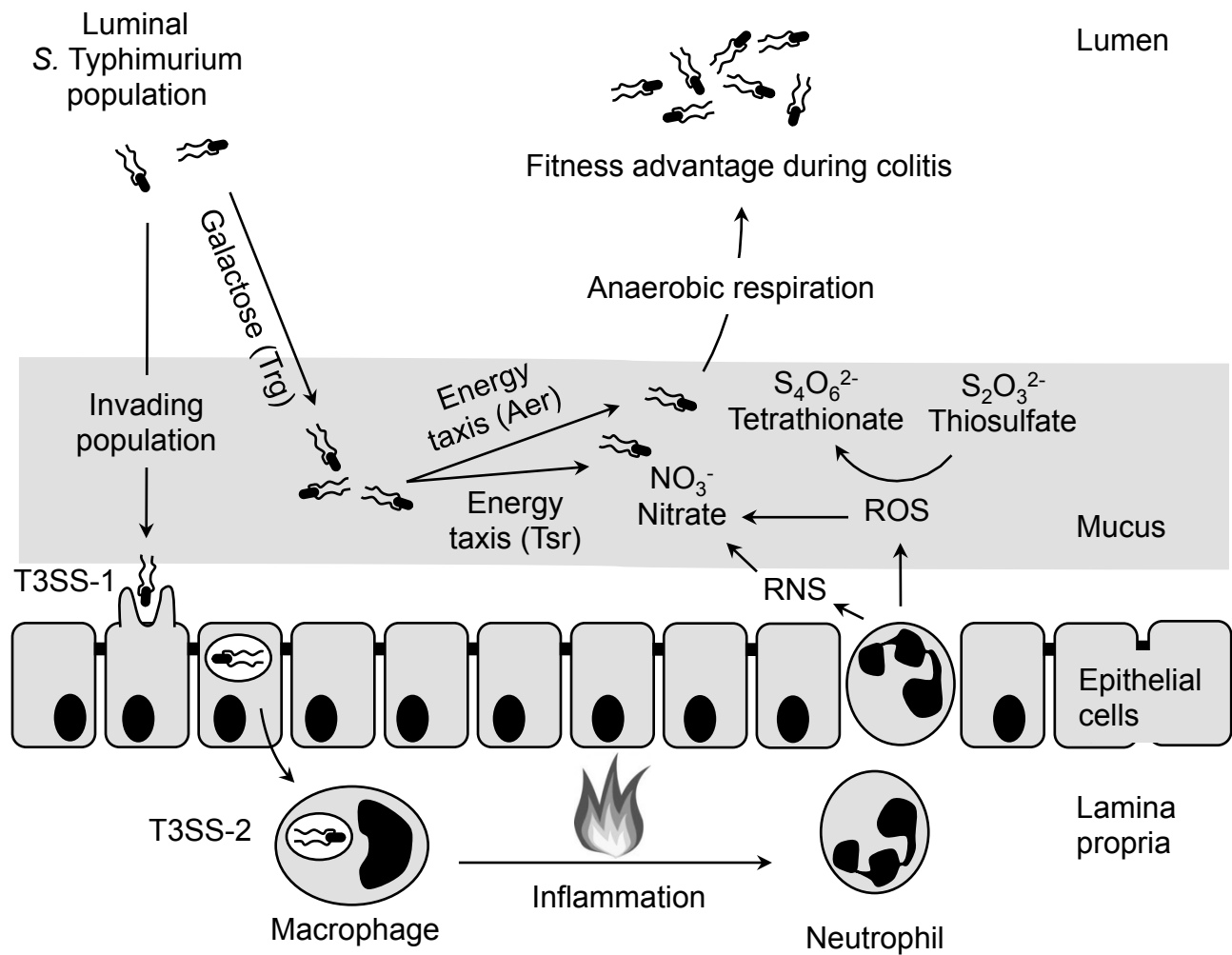

Supplement: Figure S6 — Model for the mechanism by which energy taxis confers a fitness advantage during colitis. Upon ingestion, a fraction of the luminal S. Typhimurium population migrates along a concentration gradient of galactose residues towards the mucus layer [6], but in the absence of inflammation, growth of this population is limited. Another fraction of the population uses its virulence factors to invade the intestinal epithelium (T3SS-1) and survive in macrophages (T3SS-2), which results in intestinal inflammation. Neutrophils recruited during this process transmigrate into the intestinal lumen and produce reactive oxygen species (ROS) and reactive nitrogen species (RNS) to kill bacteria. A by-product of releasing ROS and RNS is the generation of respiratory electron acceptors (tetrathionate and nitrate). The luminal fraction of the S. Typhimurium population now uses energy taxis to migrate towards environments containing tetrathionate or nitrate and subsequently uses anaerobic respiration to gain a fitness advantage over competing microbes that grow by fermentation. (PDF) [file ppat.1003267.s006.pdf]
