# Supplementary material for: Salmonella Uses Energy Taxis to Benefit from Intestinal Inflammation
Source: PLoS Pathog. 2013 Apr 18;9(4):e1003267. doi: 10.1371/journal.ppat.1003267 (PMC3630101; doi:10.1371/journal.ppat.1003267)
Supplement: Table S1 — Calculation of competitive indices for experiments using the capillary assay. S, strain number; I, inoculum; G.M, geometric mean; C/ml, colony forming units per ml; CFU/cap, geometric mean of colony forming units per capillary after incubation; IR, input ratio; OR, output ratio; CI, competitive indices; NR, napA narZ narG mutant; T (5 mM), capillary containing 5 mM tetrathionate; B, capillary containing buffer; N (1 mM), capillary containing 1 mM nitrate; (O2), experiment was performed under aerobic conditions. (PDF) [file ppat.1003267.s007.pdf]

Table S1: Calculation of competitive indices (CI) for caplillary assay

competition	S	genotype	I (G.M) C/ml	IR	CFU/cap	(OR)	CI (OR/IR)	in capillary
wt vs. *aer*	1	wt	**5.59E+07**	1.05	**2.44E+05**	1.51	1.43	T (5 mM)
	2	*aer*	**5.30E+07**		**1.62E+05**			
aer (pFR5) vs. *aer*	1	*aer* (pFR5)	**4.14E+07**	1.05	**2.94E+05**	1.57	1.49	T (5 mM)
	2	*aer*	**3.94E+07**		**1.88E+05**			
wt vs. *aer*$_{FAD}$	1	wt	**3.60E+07**	1.06	**2.71E+05**	1.70	1.61	T (5 mM)
	2	*aer*$_{FAD}$	**3.41E+07**		**1.59E+05**			
ttrA vs. *ttrA aer*	1	*ttrA*	**3.15E+07**	1.08	**8.32E+04**	1.10	1.02	T (5 mM)
	2	*ttrA aer*	**2.92E+07**		**7.54E+04**			
wt vs. *tsr*	1	wt	**3.13E+07**	0.91	**1.82E+05**	0.98	1.07	T (5 mM)
	2	*tsr*	**3.44E+07**		**1.86E+05**			
ttrA vs. *ttrA tsr*	1	*ttrA*	**3.14E+07**	0.99	**1.10E+05**	1.08	1.10	T (5 mM)
	2	*ttrA tsr*	**3.18E+07**		**1.02E+05**			
wt vs. *aer*	1	wt	**5.59E+07**	1.05	**1.05E+05**	1.03	0.98	B
	2	*aer*	**5.30E+07**		**1.02E+05**			
aer (pFR5) vs. *aer*	1	*aer* (pFR5)	**4.14E+07**	1.05	**1.29E+05**	1.08	1.02	B
	2	*aer*	**3.94E+07**		**1.20E+05**			
wt vs. *aer*$_{FAD}$	1	wt	**3.60E+07**	1.06	**1.39E+05**	1.03	0.97	B
	2	*aer*$_{FAD}$	**3.41E+07**		**1.36E+05**			
ttrA vs. *ttrA aer*	1	*ttrA*	**3.15E+07**	1.08	**6.33E+04**	0.91	0.85	B
	2	*ttrA aer*	**2.92E+07**		**6.93E+04**			
wt vs. *tsr*	1	wt	**3.13E+07**	0.91	**6.85E+04**	0.86	0.94	B
	2	*tsr*	**3.44E+07**		**8.01E+04**			
wt vs. *aer*	1	wt	**4.09E+07**	1.02	**1.07E+05**	0.83	0.81	T (5mM) (02)
	2	*aer*	**4.00E+07**		**1.28E+05**			
wt vs. *aer*	1	wt	**2.60E+07**	0.80	**2.49E+05**	1.51	1.87	N (5mM)
	2	*aer*	**3.23E+07**		**1.65E+05**			
napA narZ narG vs. napA narZ narG aer	1	*NR*	**3.35E+07**	1.20	**4.66E+04**	1.01	0.84	N (5mM)
	2	*NR aer*	**2.79E+07**		**4.61E+04**			
wt vs. *aer*	1	wt	**3.84E+07**	1.10	**3.07E+05**	1.32	1.21	N (1 mM)
	2	*aer*	**3.51E+07**		**2.32E+05**			
wt vs. *tsr*	1	wt	**3.56E+07**	0.99	**3.25E+05**	1.03	1.04	N (1 mM)
	2	*tsr*	**3.59E+07**		**3.17E+05**			
wt vs. *aer*	1	wt	**3.84E+07**	1.10	**2.75E+05**	0.87	0.80	N (0.1 mM)
	2	*aer*	**3.51E+07**		**3.15E+05**			
wt vs. *tsr*	1	wt	**3.56E+07**	0.99	**3.32E+05**	2.19	2.21	N (0.1 mM)
	2	*tsr*	**3.59E+07**		**1.52E+05**			
tsr (pFR6) vs. *tsr*	1	*tsr (pFR6)*	**4.91E+07**	0.98	**3.46E+05**	2.12	2.16	N (0.1 mM)
	2	*tsr*	**5.00E+07**		**1.63E+05**			
napA narZ narG vs. napA narZ narG tsr	1	*NR*	**3.65E+07**	1.08	**4.56E+04**	1.00	0.93	N (0.1 mM)
	2	*NR aer*	**3.38E+07**		**4.55E+04**			
wt vs. *aer*	1	wt	**3.84E+07**	1.10	**2.55E+05**	0.88	0.80	N (0.1 mM)
	2	*aer*	**3.51E+07**		**2.90E+05**			
wt vs. *tsr*	1	wt	**3.56E+07**	0.99	**2.89E+05**	1.23	1.24	N (0.1 mM)
	2	*tsr*	**3.59E+07**		**2.35E+05**			
wt vs. *aer*	1	wt	**2.60E+07**	0.80	**6.07E+04**	0.77	0.95	B
	2	*aer*	**3.23E+07**		**7.93E+04**			
wt vs. *tsr*	1	wt	**3.56E+07**	0.99	**1.29E+05**	0.99	1.00	B
	2	*tsr*	**3.59E+07**		**1.30E+05**			

Table S1: Calculation of competitive indices (CI) for caplillary assay

tsr (pFR6) vs. *tsr*	1	*tsr (pFR6)*	**4.91E+07**	0.98	**1.34E+05**	0.99	1.01	B
	2	*tsr*	**5.00E+07**		**1.35E+05**			
wt vs. *aer*	1	wt	**4.51E+07**	1.11	**5.62E+04**	0.86	0.77	N (5 Mm) (02)
	2	*aer*	**4.08E+07**		**6.56E+04**			
wt vs. *tsr*	1	wt	**3.56E+07**	0.99	**1.17E+05**	**0.88**	0.89	N (0.1 mM) (02)
	2	*tsr*	**3.59E+07**		**1.34E+05**			
